# Antimicrobial Susceptibility and Genomic Analysis of *Aliarcobacter cibarius* and *Aliarcobacter thereius*, Two Rarely Detected *Aliarcobacter* Species

**DOI:** 10.3389/fcimb.2021.532989

**Published:** 2021-03-17

**Authors:** Ingrid Hänel, Eva Müller, Belén González Santamarina, Herbert Tomaso, Helmut Hotzel, Anne Busch

**Affiliations:** ^1^ IBIZ, Friedrich-Loeffler-Institut Jena, Jena, Germany; ^2^ Department of Anaesthesiology and Intensive Care Medicine, University Hospital Jena, Jena, Germany

**Keywords:** *Aliarcobacter*, genomic analysis, antibiotic susceptibility, ciprofloxacin resistance, sequencing

## Abstract

*Aliarcobacter cibarius* and *Aliarcobacter thereius* are two rarely detected *Aliarcobacter* species. In the study, we analyzed the antimicrobial susceptibility and provide detailed insights into the genotype and phylogeny of both species using whole-genome sequencing. Thermophilic *Campylobacter* species are the most common bacterial foodborne pathogens causing gastroenteritis in humans worldwide. The genus *Aliarcobacter* is part of the *Campylobacteraceae* family and includes the species *Aliarcobacter butzleri, Aliarcobacter cryaerophilus, Aliarcobacter skirrowii*, and the rarely described *Aliarcobacter cibarius, Aliarcobacter faecis, Aliarcobacter lanthieri, Aliarcobacter thereius*, and *Acrobarter trophiarum. Aliarcobacter* are emergent enteropathogens and potential zoonotic agents. Here, we generated, analyzed, and characterized whole-genome sequences of *Aliarcobacter cibarius* and *Aliarcobacter thereius.* They were isolated from water poultry farms in Germany, cultured and identified by MALDI-TOF MS. With PCR the identity was verified. Antibiotic susceptibility testing was carried out with erythromycin, ciprofloxacin, doxycycline, tetracycline, gentamicin, streptomycin, ampicillin, and cefotaxime using the gradient strip method (E-test). Whole-genome sequences were generated including those of reference strains. Complete genomes for six selected strains are reported. These provide detailed insights into the genotype. With these, we predicted *in silico* known AMR genes, virulence-associated genes, and plasmid replicons. Phenotypic analysis of resistance showed differences between the presence of resistance genes and the prediction of phenotypic resistance profiles. In *Aliarcobacter butzleri*, the nucleotide sequence of the *gyr*A gene (DQ464331) can show a signature mutation resulting in an amino acid change T85>I. *Acrobarter cibarius* and *Acrobarter thereius* showed the same gene as assessed by similarity annotation of the mutations 254C>G. Most of the isolates were found to be sensitive to ciprofloxacin. The ciprofloxacin-resistant *Aliarcobacter thereius* isolate was associated with the amino acid change T85>I. But this was not predicted with antibiotic resistance databases, before. Ultimately, a phylogenetic analysis was done to facilitate in future outbreak analysis.

## Introduction

Thermophilic *Campylobacter* (*C*.) are the most common bacterial cause of human gastroenteritis in the world (WHO, 2018). The genus *Aliarcobacter* (*A*.) within the family *Campylobacteriaceae* was created to accommodate Gram-negative, curved-shaped bacteria belonging to atypical *Campylobacter* spp. classified by their ability to grow at lower temperatures (15–30°C) and without microaerophilic conditions (Vandamme et al., 1991). The genus *Aliarcobacter* (formerly *Arcobacter*) consists of *A. butzleri*, *A. cibarius*, *A. cryaerophilus*, *A. faecis*, *A. lanthieri*, *A. skirrowii*, *A. thereius* and *A. trophiarum* (Pérez-Cataluña et al., 2017; Pérez-Cataluña et al., 2018a; Pérez-Cataluña et al., 2018b; Pérez-Cataluña et al., 2018c). *Aliarcobacter* spp. are present in the digestive tract of healthy animals (Uenver et al., 2013), but have also been associated with enteritis and reproductive disorders (De Smet et al., 2012). *Aliarcobacter* in contaminated food (*e.g.* poultry products) and water can be transmitted to humans and can cause diarrhea and in rare cases bacteremia (Collado and Figueras, 2011; Hanel et al., 2016).

The species *A. butzleri*, *A. cryaerophilus*, and *A. skirrowii* are considered emergent enteropathogens and potential zoonotic agents (Prouzet-Mauleon et al., 2006; Levican et al., 2013; Van den Abeele et al., 2014; Van den Abeele et al., 2016; Hänel et al., 2018). *A. cibarius* and *A. thereius* are present in farm animals and have been isolated from food of animal origin (Chinivasagam et al., 2007; Pentimalli et al., 2009; Adam et al., 2014a; Yesilmen et al., 2014) but have not yet been isolated from human specimens. The pathogenicity and virulence mechanisms of *Aliarcobacter* spp. are still poorly understood. Adhesion, invasion, and cytotoxicity capacity have been studied only in *A. skirrowii, A. butzleri*, *A. cryaerophilus*, and *A. cibarius* (Collado and Figueras, 2011; Pérez-Cataluña et al., 2018b), and isolates have been rarely sequenced.

Isolation and taxonomic identification of *A. cibarius* and *A. thereius* are often difficult. Only a few representative genomes have been described so far (Adam et al., 2014a; Adam et al., 2014b; Rovetto et al., 2017). There are few accessible data providing the antibiotic susceptibility of *A. cibarius* and *A. thereius* (Abdelbaqi et al., 2007; Fanelli et al., 2019).

The aim of this study was to determine the antimicrobial susceptibility of *A. cibarius* and *A. thereius* isolated from domestic water poultry to antibiotics commonly used in treatments of humans. Additionally, the genomic features of *A. cibarius* and *A. thereius* isolates were analyzed to improve diagnostic and antibiotic treatment options. The study presents a full genome analysis of *A. cibarius* and *A. thereius* isolates and their reference strains, available from a public culture collection. The genomes of reference strains were analyzed with a focus on virulence-associated genes and antibiotic resistance genes. The strains were analyzed for the *gyr*A gene known to be causal for ciprofloxacin resistance in *A*. *butzleri.* Data were compared to the *A. butzleri* strain D2686 DNA gyrase subunit A gene (DQ464331) (Abdelbaqi et al., 2007). Subsequently, phylogenetic strain variation in *A. cibarius* and *A. thereius* was assessed to analyze the previously reported high heterogeneity (De Smet et al., 2012; Pérez-Cataluña et al., 2018b).

## Methods

### Strain Isolation and Identification

The *A. cibarius* and *A. thereius* isolates were handled as before reported for *A. skirrowii* (Hänel et al., 2018). *Aliarcobacter* isolates were cultivated from fecal samples collected in two water poultry farms in Thuringia, Germany. A two-step procedure was done in *Arcobacter* broth (Oxoid GmbH, Wesel, Germany) supplemented with antibiotics cefoperazone, amphotericin, and teicoplanin (CAT; Oxoid GmbH) under microaerophilic conditions (5% O_2,_ 10% CO_2_ and 85% N_2_) for 48 h at 30°C. Subsequently, the broth was streaked on plates (Mueller-Hinton agar/CAT/5 % defibrinated bovine blood) and incubated under microaerophilic conditions for another 24–48 h at 30°C. Suspicious colonies were recultivated and identified by matrix-assisted laser desorption/ionization time-of-flight mass spectrometry (MALDI-TOF MS) as described before (El-Ashker et al., 2015; Busch et al., 2018). IVD Bacterial Test Standard, Biotyper 3.1 software and the database DB 4613 (all Bruker Daltonik GmbH, Bremen, Germany) containing spectra of all *Aliarcobacter* species were used. Confirmation of the species identification was performed with multiplex PCR as described before (Hänel et al., 2018).

Sequence data used in this study had the following accession numbers: *A. anaerophilus* PDKO01*, A. butzleri* RM4018, CP000361.1*, A. halophilus* DSM 18005, GenBank: NXIF00000000.*, A. aquimarinus* CECT 8442, NCBI Reference Sequence NZ_NXIJ00000000.1, *A. bivalviorum* LMG 26154 NZ_CP031217.1, *A. cibarius* LMG 21996, NCBI Reference Sequence: NZ_JABW00000000.1, *A. cloacae* CECT 7834, NZ_NXII00000000.1, *A. cryaerophilus* L406, NZ_LRUV00000000.1.

### Antibiotic Susceptibility Testing

Antimicrobial susceptibility to eight antibiotics (erythromycin, ciprofloxacin, doxycycline, tetracycline, gentamicin, streptomycin, ampicillin, and cefotaxime) was determined using the gradient strip diffusion method (E-test™, bioMérieux, Nürtingen, Germany) following the instructions (**Table 1**). The bacterial suspensions for the E-test were adjusted to an optical density of 0.1 at 600 nm (approximately 3 to 5 × 10^8^ cfu/ml) in phosphate-buffered saline (PBS). 750 µl was spread on a Mueller-Hinton agar plate and a single strip was put on each plate. After 48 h of incubation at 30°C under microaerophilic conditions, the minimum inhibitory concentration (MIC) was determined. The type strain of *A. skirrowii* DSM 7302 was used as control. For erythromycin, ciprofloxacin, doxycycline, and tetracycline, interpretative criteria were based upon EUCAST breakpoints for *Campylobacter* (2019). For gentamicin, ampicillin, and cefotaxime (EUCAST, 2019) *Enterobacterales* breakpoints were applied (2019). For streptomycin, the cut-off value for *C. jejuni* was used as suggested by the EFSA–working group (EFSA, 2008). The phenotypes were classified as sensitive (S), and resistant (R) (**Table 1**).

**Table 1:** Antibiotic susceptibility of *Arcobacter* spp. EUCAST breakpoints for *Campylobacter jejuni* was used for erythromycin, ciprofloxacin, doxycycline, tetracycline, and gentamicin.

	DSM 17680		16CS0831-2		16CS0831-3		DSM 23385		17CS1191		17CS1200	
	*Arcobacter cibarius*		*Arcobacter cibarius*		*Arcobacter cibarius*		*Arcobacter thereius*		*Arcobacter thereius*		*Arcobacter thereius*	
	*Reference isolate*		Thuringia, Farm 1/goose		Thuringia, Farm 1/goose		Reference isolate		Thuringia, Farm 2/duck		Thuringia, Farm 1/duck	
	1	2	1	2	1	2	1	2	1	2	1	2
Erythromycin S ≤4 mg/L R >4 mg/L	S 2.0	No	S 3.0	No	S 4.0	No	S 3.0	No	S 1.0	No	S 2.0	No
Ciprofloxacin S ≤0.5 mg/L R >0.5 mg/L	S 0.12	No	S 0.12	No	S 0,12	No	S 0.75	No	S 0.25	No	**R** **>32**	No
Doxycycline S ≤2 mg/L R >2 mg/L	S 0.5	No	S 0.5	No	S 0.75	No	S 0.36	No	S 0.38	No	S 1.25	No
Tetracycline S ≤2 mg/L R >2 mg/L	S 0.5	No	S 0.5	No	S 0.5	No	S 1.0	No	S 0.5	No	S 0.9	No
Gentamicin S ≤2 mg/L R >4 mg/L	S 0.5	No	S 0.5	No	S 0.75	No	S 1.5	No	S 1.0	No	S 3.5	No
Streptomycin S ≤2 mg/L R >2 mg/L	R 1.5	No	S 1.5	No	S 1.5	No	R 14	No	R 8	No	R 8	No
Ampicillin	S	No	S	No	S	No	S	No	S	No	S	No
Cefotaxime	**R**	No	**R**	No	**R**	No	**R**	No	**R**	No	**R**	No

EFSA breakpoints were applied for streptomycin (EFSA, 2008). S, susceptible; R, resistant; 1 = Laboratory results with E-test; 2 = ResFinder prediction.

Resistances were highlighted in bold.

### Sequencing and Genomic Analysis

DNA for whole-genome sequencing (WGS) was prepared from colonies harvested from plates. DNA was purified (High Pure PCR Template Preparation Kit; Roche Diagnostics, Mannheim, Germany) and sequencing libraries were generated using the Nextera XT DNA Library Prep Kit (Illumina, Inc., San Diego, CA, USA). From an Illumina MiSeq run paired-end reads were generated to mean sequencing depth between 50 and 100 reads. The assignment of the taxonomic labels to all reads was performed with MetaPhlAn (Segata et al., 2012) and Kraken version 0.10.6 (Wood and Salzberg, 2014). Further, read processing included quality trimming and assembly with SPAdes v3.12.0 (–careful) (Bankevich et al., 2012), and filtering by removing contigs with a coverage <5 and a length <500. Annotation was performed with Prokka using the recommended standard settings (Seemann, 2014). The report of the presence of antibiotic resistance genes *in silico* was compared with results from *in vivo* susceptibility testing.

### Assignment of Phylogeny, Resistance, Plasmids, and Virulence-Associated Genes and MLST

**Figure 1:**
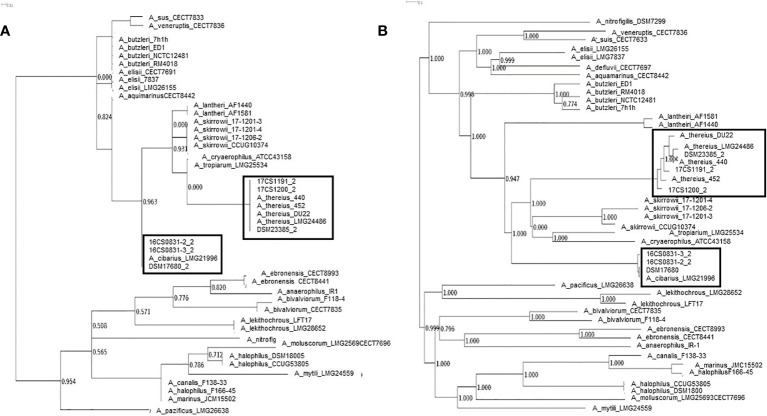
Phylogeny based on all identified SNPs and phylogenetic analysis without genome alignment or the requirement for reference genomes all SNPs and reference independent performed with kSNP3.0. **(A)** Core genome analysis and **(B)** parsimony tree based on whole-genome SNPs.

Additionally, parSNP and kSNP3.0 (Treangen et al., 2014; Gardner et al., 2015) were used to assign microbial phylogeny and results were visualized with Dendroscope (Huson et al., 2007) (**Figure 1**). Based on ARIBA (Hunt et al., 2017) several databases were used to identify single nucleotide polymorphisms (SNPs) directly from short reads. For detection of resistance genes the ResFinder (Zankari et al., 2012) and for plasmid finding the PlasmidFinder (Carattoli et al., 2014) were used. Multilocus Sequence Typing (MLST) was done using the MLST database (Carattoli et al., 2014) and for detection of virulence factors VFDB_full was used (Chen et al., 2005) (**Table 2**). Virulence-associated genes *cia*B (HF935951)*, cj*1349 *(*HF935963), and *cad*F (HF935942) were mapped with Geneious (Kearse et al., 2012) on all assemblies and to the known *A. skirrowii* sequence LRUX01000036.1. All the reads of all isolates were mapped to a set of six putative virulence-associated genes (*cia*B, *pld*A, *tly*A, *mvi*N, *cad*F, and *cj*1349) (Karadas et al., 2013; Douidah et al., 2014; Sekhar et al., 2017). Further analysis of plasmids was done with Bandage (Wick et al., 2015). Reads were mapped against selected antibiotic genes known to be responsible to resistance to ampicillin and cefotaxime. The following genes were selected from databases with the following locus tags: A7H1H_0053-16806891, A7H1H_1295-16807124, A7H1H_1494-16808301, ABED_1213-12128208, ABED_1379-12128538, Abu_0578-5624058, Abu_1299-5623980, Abu_1478-5624289, *bla*OXA-24305112, CHAB381_1693-5408367, *cj*0299-904623, *cme*D-12249850, *cme*E-12249851, *cme*F-12249852, *fts*I-16808465, *pbp*C-5624113, *pbp*C-16807438, ABU_RS00920-24304134.

**Table 2:** Assembly data of *Aliarcobacter cibarius* and *Arcobacter thereius* isolates.

Assembly	16CS0831-2_2	16CS0831-3_2	DSM17680_2	17CS1191_2	17CS1200_2	DSM23385_2
Number of contigs	78	77	77	11	19	22
Largest contig	186,593	186,571	298,255	512,801	488,536	392,917
Total length	2,233,676	2,233,860	2,157,340	1,930,425	1,873,208	1,897,659
GC (%)	26.87	26.87	26.77	26.79	26.84	26.86
N50	68,388	63,497	121,612	396,252	448,176	170,586
Mean coverage	102	136	133	126	111	116
Total reads	776,586	1,173,342	993,276	941,318	842,376	990,376

## Results

*A. cibarius* and *A. thereius* isolates were cultivated and identified by MALDI-TOF MS due to scores >2,3 (spectra are available upon request). Identification was confirmed by PCR. Taxonomic analysis of the WGS data with MetaPhlAn and Kraken resulted for *A. cibarius* and *A. thereius* in “unclassified *Arcobacter”*. Also, analyses based on the genomic features by sequence identity with RAST, Rapid Annotation using Subsystem Technology, (Aziz et al., 2008) showed only a relationship to *A. butzleri* RM4018 for all isolates. A correct taxonomic differentiation of the species with the reference strains was possible with SNP detection using parSNP and kSNP 3.0.

The phenotypic antibiotic susceptibility resulting by E-test for the *Aliarcobacter* isolates is shown in **Table 1**. All *A. cibarius* and *A. thereius* isolates were susceptible to erythromycin, doxycycline, tetracycline, streptomycin, ampicillin, and gentamicin and showed resistance to cefotaxime. The isolate *A. thereius* 17CS1200 was resistant to ciprofloxacin. No resistance determinants were predicted with the ResFinder (see **Table 1**).

The mean coverage using WGS obtained for the isolates was >100 (see **Table 3**). All sequences were assembled and annotated. Genome assemblies had 11 to 78 contigs. Sequence length prediction was 1,897,659–2,233,860 bases. All assemblies had a mean coverage of 102–136 resulting in high-quality genome drafts. The genome sizes were estimated between 1.9–2.2 million bases with approximately 1,980 coding sequences. The programs as used in Busch et al. (Busch et al., 2020a; Busch et al., 2020b) were applied with standard settings. No reads mapped to reference genes in the database, therefore, no local assemblies were run and no SNPs were detected. However, the known virulence-associated genes *cj*1349 *(*HF935963), *cia*B (HF935951)*, cad*F (HF935942) and *hec*A (HF935064) were found and could be mapped to all assemblies as in the published *A. skirrowii* sequence LRUX01000036.1 (Hänel et al., 2018) (**Table 3**). However, all genes mapped with a low sequence identity (49.9 to 87.0%). For the ampicillin and cefotaxime genes, the following genes could be mapped with more than 95% identity: A7H1H_0053-16806891, A7H1H_1295-16807124, *fts*I-16808465, *pbp*C-5624113, *pbp*C-16807438, and AA347_RS09755-39300940. No mapping was detected with assemblies with the exclusion of AA347_RS01370-39299273 deriving from *A. thereius* LMG24486. From the sequencing data, MLST results were extracted. Sequence types were assigned (**Supplementary Table 3**). The results were not verified by traditional sequencing methods for MLST. As alternative method phylogenetic analysis was done (**Figure 1**). The first approach was based on all identified SNPs of the core genome (Treangen et al., 2014). The second approach was done without reference genome alignment kSNP3.0 (Gardner et al., 2015) with subsequent parsimony tree based on all SNPs detected in the genomes. Both approaches confirmed the classification of the isolates obtained by MALDI-TOF MS and PCR and proofed as suitable tools. Outbreak analysis, diagnostics and characterization of unknown isolates can be done with these methods.

**Table 3:** Reads of all *Aliarcobacter* spp. mapped to known virulence-associated genes of *Arcobacter butzleri* RM4018 (Karadas et al., 2013).

Genes annotated to *A. butzleri* RM4018	DSM 17680	16CS0831-2	16CS0831-3	DSM 23385	17CS1191	17CS1200
*cia*B—5623903, invasion protein CiaB CDS	+	+	−	+	+	+
*mvi*N—5625241, murein biosynthesis integral membrane protein MurJ CDS	+	+	−	+	+	+
*cad*F—5623687, OmpA family protein CDS	+	+	−	−	−	−
*pld*A—5625169, phospholipase A CDS	−	+	−	−	−	−
*tly*A—5623177, TlyA family RNA methyltransferase CDS	+	+	−	+	+	+

Complete Coverage (+) or no or partial coverage (−).

## Discussion

*A. butzleri*, *A. cryaerophilus*, and *A. skirrowii* have been associated with gastrointestinal diseases. For *A. cibarius* and *A. thereius*, pathogenicity is unknown (Collado and Figueras, 2011; Isidro et al., 2020). It can be assumed that *Aliarcobacter* infections in human are so far underestimated. Thus, we assessed genomic and phenotypic characteristics of the rare species *A. cibarius* and *A. thereius.* Here, we present cultivation protocols for *A. cibarius* and *A. thereius*. We evaluated MALDI-TOF MS spectra and made them available. Genome sequencing data were published at the NCBI so that the software tools based on the RefSeq (such as BLAST and Kraken) will allow quick identification in the future.

Data on antimicrobial susceptibility of *A. cibarius* and *A. thereius* are scarce. *A. cibarius* and *A. thereius* are reported mostly susceptible to many antimicrobials (Houf et al., 2001; Yesilmen et al., 2014) in concordance with the hereby reported isolates. Antibiotic testing is not standardized, yet. Microdilution assays are usually favored, but gradient strips are more robust (Van den Abeele et al., 2014; Hänel et al., 2018). *A. butzleri* or *A. cryaerophilus* isolates from Europe showed susceptibility to gentamicin, tetracycline, erythromycin, ciprofloxacin, and doxycycline and some of them resistance to ampicillin (Van den Abeele et al., 2016). Phenotypic analysis of resistance showed differences between the prediction of resistance genes and the phenotypic resistance profile. The here investigated *A. cibarius* and *A. thereius* isolates were mostly sensitive to ciprofloxacin but the isolate 17CS1200 was resistant. This could not be predicted with antibiotic resistance databases, ResFinder (Zankari et al., 2012), yet. Resistance to ciprofloxacin is reported for almost 25 % of human *Campylobacter* isolates (CDC, 2015). In *A. butzleri*, the nucleotide sequence of the *gyr*A gene (DQ464331) has the signature mutation resulting in an amino acid change T85>I (Abdelbaqi et al., 2007). The isolates of *A. cibarius* and *A. thereius* that were sensitive to ciprofloxacin showed a nucleotide change 254C>G that resulted in T85>S changing on protein basis. The ciprofloxacin-resistant isolate 17CS1200 had an amino acid change T85>I. This is the described signature mutation as reported in *A. butzleri*. It can be assumed that this, as a universally functional mechanism, is likely to affect DNA supercoiling, and the expression of several virulence factors and proteins as described in different species is the same (Ozeki et al., 1997; Aubry et al., 2006; Abdelbaqi et al., 2007; Hashimi et al., 2008; Lau et al., 2011; Malik et al., 2012; Yamachika et al., 2012; Sutera et al., 2017). Ciprofloxacin resistance usually occurs due to specific point mutations within the DNA gyrase A (*gyrA)* and/or topoisomerase IV *parC* and *parE* genes, often in combination (Casin et al., 2003; Johnning et al., 2015; Onseedaeng and Ratthawongjirakul, 2016). Annotation of mutation in the gyrase A gene is considered not to be sufficient to produce resistance to ciprofloxacin (Hooper and Jacoby, 2015). For example mutations in *gyr*A and *par*E subunits of the respective enzymes can reduce drug-binding to the enzyme–DNA complex. But also plasmid-encoded resistance due to Qnr proteins can shield from quinolone action. On the other hand, single mutations in *gyr*A are known to generate resistance to nalidixic acid, a first-generation quinolone. However, additional mutations in other type II topoisomerase genes are often necessary to generate resistance to later generations of fluoroquinolones, such as ciprofloxacin (Johnning et al., 2015). A mutation as in the *par*E gene as in *Salmonella* could not be detected.

The antibiotic susceptibility results we report here are in agreement with antibiotic resistance or susceptibility prevalence reported for other *Aliarcobacter* isolates from seafood and water sources (Levican et al., 2013; Levican et al., 2014; Levican et al., 2015; Rathlavath et al., 2017a; Rathlavath et al., 2017b; Silha et al., 2017). *Aliarcobacter* resistance is reported in food sources as meat, milk, shellfish, slaughterhouses, dairy plants, and humans, although rarely done for rare *Aliarcobacter* comparing phenotype and genotype (Atabay et al., 2008; Ferreira et al., 2013; Ferreira et al., 2014a; Ferreira et al., 2014b; Ferreira et al., 2016; Shirzad Aski et al., 2016; Ferreira et al., 2017; Ferreira et al., 2018; Isidro et al., 2020).

In *Campylobacter* infections macrolides are used as preferred therapeutic agents. Tetracycline was proposed for severe cases only (Yan et al., 2000; Kayman et al., 2012; Arguello et al., 2015). Here investigated isolates were susceptible to both classes of antibiotics and would guarantee effectiveness in treatment of infections. Monitoring and reporting of antimicrobial resistance data even in rare species, as well as whole-genome sequencing data analysis of *Aliarcobacter* from domestic animals are important to monitor the evolution of antimicrobial resistance. This can be used to optimize diagnostics and therapy.

Gene transfer or plasmid transfer events can induce antibiotic resistance (Sundin and Bender, 1996). In the *Enterobacteriaceae* family, antibiotic resistance is often mediated by plasmids (Nikaido, 2009). Indication is here that the antimicrobial resistance determinants are located on the chromosome. Plasmids play an important role in the genetic evolution and adaptation. In *A. butzleri* 9.9% of isolates carried plasmids (Harrass et al., 1998; Toh et al., 2011). PlasmidFinder could not predict plasmids nor plasmids could be detected by the graphical assembly graphs, allowing visual inspection (Wick et al., 2015). In agarose gels, no plasmids could be detected in *A. cibarius* and *A. thereius* comparable with *A. skirrowii* (Becker et al., 2016; Hänel et al., 2018).

MLST characterizes isolates using allelic DNA sequences of several housekeeping genes and is a traditional technique. Here, no MLST genes could be predicted for both rarely found *Aliarcobacter* species. As a consequence, MLST might be replaced by phylogeny based on all identified SNPs of the core or whole genomes.

Virulence factors were not predicted. But genes like *cia*B, *pld*A, *tly*A, *mvi*N, *cad*F, and *cj*1349 are known to be involved in host adherence and invasion. They could be mapped with low sequence identity (Levican et al., 2013). The protein CiaB has been shown to be responsible for host cell invasion in *Campylobacter.* The protein TlyA is a hemolysin with a role in cellular adherence in *C. jejuni.* The protein CadF and *cj1349* are fibronectin-binding proteins, that promote bacteria to cell contact and PldA is a phospholipase associated with lysis of erythrocytes (Miller et al., 2007; Sekhar et al., 2017; Isidro et al., 2020). The low sequence identity was also an indication that *A. cibarius* and *A. thereius* might show more variability within the nucleotide sequences. This will benefit to be included the databases.

As a suitable tool, we evaluated whole-genome sequencing with phylogenetic analysis. A parsimony tree analysis based on SNPs in the genomes proofed to be specifically useful. With these outbreak analysis, diagnostics and characterization can be approached even for rare species and classify new, not yet unclassified species correctly. A phylogenetic tree revealed the relatedness of these isolates and will facilitate in future outbreak analysis. With the provided genomes a core genome analysis and whole-genome SNP phylogeny analysis provided exact classification of identified strains and reference strains. This phylogenetic analysis has the advantage that it is reference-free and alignment-independent. This allows for the usage of the method for the previously reported high heterogeneity of isolates and a correct placement within the phylogenetic tree, even when the strains are previously unknown (De Smet et al., 2012; Pérez-Cataluña et al., 2018b; Pérez-Cataluña et al., 2019).

The application of open-source software allows an economic and transparent assessment of the sequencing data. The diagnostic capabilities can be improved and contributes to epidemiological, pathogenic and functional analysis of these rarely recognized bacteria. The mechanisms underlying the pathogenicity of *Aliarcobacter* may thus be further elucidated. The optimized database will serve bioinformatics tools to do comprehensive bioinformatics analysis. It remains speculative that the eradication of *Campylobacter* spp. from the microbiome of farmed animals could foster the replacement by *Aliarcobacter* subspecies. In such case, any basic assessment of the pathogenicity of *Aliarcobacter* spp. will be advantageous.

In conclusion, *A. cibarius* and *A. thereius* are rarely detected but potentially harmful bacteria. Due to the fastidious growth of *A. cibarius* and *A. thereius* the antibiotic susceptibility testing is preferably done using the gradient strip method. Prediction of antibiotic susceptibility, plasmid sequences, MLST or virulence factors based on WGS data should be carefully assessed. The present study adds the aspect of antimicrobial phenotyping of the *A. cibarius* and *A. thereius* species into *Aliarcobacter* comparative genomics field. In fact, we here provide extended data on core and full genome phylogenetics and some gene-by-gene assignment of these bacteria showing that further functional and expressional studies are needed to understand the genotypic and phenotypic correlations of genomes and genes. Our data supports the notion that some aspects of the virulome and resistome diversity, environmental/host adaptation and pathogenicity need to be adapted for rare species. This is highlighting the need to implement more robust species-oriented diagnostics towards an enhanced global surveillance and control of this emerging pathogen.

## Data Availability Statement

The datasets generated for this study can be found in the Data availability: *Arcobacter cibarius* and *Arcobacter thereius* sequences were deposited at BioProject under the accession number PRJNA542783, and as BioSample under the accession number SAMN11638312 (VBUC00000000, *Arcobacter cibarius* 16CS0831-2), SAMN11638336 (VBUD00000000, *Arcobacter cibarius* 16CS0831-3), SAMN11638387 (VBUE00000000, *Arcobacter cibarius* DSM17680), SAMN11638388 (VBUF00000000, *Arcobacter thereius* 17CS1191_2), SAMN11638390 (VBUG00000000, *Arcobacter thereius* 17CS1200_2), SAMN11638433 (VBUH00000000, *Arcobacter thereius* DSM23385). The version described in this paper is the first version.

## Author Contributions

IH, HH, BGS, AB and HT have jointly conceived the study. IH provided strains, strain information, and metadata and antibiotic testing to the samples. EM provided further antibiotic testing to the samples and bioinformatics analysis. AB performed bioinformatics analysis of genomes, assembly, and phylogenetic relationships. All authors contributed to the article and approved the submitted version.

## Funding

For this work, IH and EM were supported by an in-house project of Friedrich-Loeffler-Institut. AB was supported by a grant of the German Federal Ministry of Education and Research within the framework of the project Ess-B.A.R. (FKZ 13N13983) and by the Deutsche Forschungsgemeinschaft (German Research Foundation, DFG) under Germany’s Excellence Strategy–EXC 2051–Project-ID 390713860.

## Conflict of Interest

The authors declare that the research was conducted in the absence of any commercial or financial relationships that could be construed as a potential conflict of interest.
