# Supplementary material for: Antimicrobial Susceptibility and Genomic Analysis of Aliarcobacter cibarius and Aliarcobacter thereius, Two Rarely Detected Aliarcobacter Species
Source: Front Cell Infect Microbiol. 2021 Mar 17;11:532989. doi: 10.3389/fcimb.2021.532989 (PMC8010192; doi:10.3389/fcimb.2021.532989)
Supplement: Supplementary file 1 [file Table_1.docx]

Supplementary Table 1: Strains and accession numbers of the available genomes, selected as previously described (Pérez-Cataluña et al., 2018)

| Filename | Description | Strain | Source |
| --- | --- | --- | --- |
| 16CS0831-2_2.fasta | *Arcobacter cibarius* | 16CS0831-2 | Goose feces |
| 16CS0831-3_2.fasta | *Arcobacter cibarius* | 16CS0831-3 | Goose feces |
| 17CS1191_2.fasta | *Arcobacter thereius* | 17CS1191 | Duck feces |
| 17CS1200_2.fasta | *Arcobacter thereius* | 17CS1200 | Duck feces |
| DSM17680_2.fasta | *Arcobacter cibarius*, reference strain | DSM17680 |  |
| DSM23385_2.fasta | *Arcobacter thereius,* reference strain | DSM23385 |  |
| Arcobacter_anaerophilus_strainIR-1.fasta | *Arcobacter anaerophilus* |  |  |
| Arcobacter_aquimarinusCECT8442.fasta | *Arcobacter aquimarinus* CECT 8442 |  |  |
| Arcobacter_bivalviorumCECT7835.fasta | *Arcobacter bivalviorum* CECT 7835 |  |  |
| Arcobacter_bivalviorumF118-4.fasta | *Arcobacter bivalviorum* F118-4 |  |  |
| Arcobacter_butzleri7h1h.fasta | *Arcobacter butzleri* 7h1h, complete genome |  |  |
| Arcobacter_butzleri_RM4018.fasta | *Arcobacter butzleri* RM4018, complete genome |  |  |
| Arcobacter_butzleriED-1.fasta | *Arcobacter butzleri* ED-1 DNA, complete genome |  |  |
| Arcobacter_butzleriNCTC12481.fasta | *Arcobacter butzleri* strain NCTC12481 genome assembly, chromosome: 1 |  |  |
| Arcobacter_canalisF138-33.fasta | *Arcobacter canalis* |  |  |
| Arcobacter_cibariusLMG_21996.fasta | *Arcobacter cibarius* |  |  |
| Arcobacter_cryaerophilusATCC43158.fasta | *Arcobacter cryaerophilus* ATCC 43158 chromosome, complete genome |  |  |
| Arcobacter_defluviiCECT7697.fasta | *Arcobacter defluvii* |  |  |
| Arcobacter_ebronensis_strainCECT8993.fasta | *Arcobacter ebronensis* |  |  |
| Arcobacter_ebronensisCECT8441.fasta | *Arcobacter ebronensis* |  |  |
| Arcobacter_ebronensisCECT8993.fasta | *Arcobacter ebronensis* |  |  |
| Arcobacter_ellisii_strainLMG26155.fasta | *Arcobacter ellisii* strain LMG 26155 chromosome, complete genome |  |  |
| Arcobacter_ellisiiCECT7837.fasta | *Arcobacter ellisii* |  |  |
| Arcobacter_halophilus_strainCCUG53805.fasta | *Arcobacter halophilus* strain CCUG 53805 chromosome, complete genome |  |  |
| Arcobacter_halophilusDSM18005.fasta | *Arcobacter halophilus* |  |  |
| Arcobacter_halophilusF166-45.fasta | *Arcobacter halophilus* |  |  |
| Arcobacter_lanthieriAF1440.fasta | *Arcobacter lanthieri* |  |  |
| Arcobacter_lanthieriAF1581.fasta | *Arcobacter lanthieri* AF 1581 |  |  |
| Arcobacter_lekithochrous_strainLFT17.fasta | *Arcobacter lekithochrous* strain LFT17 |  |  |
| Arcobacter_lekithochrous_strainLMG28652.fasta | *Arcobacter lekithochrous* |  |  |
| Arcobacter_lekithochrousLMG28652.fasta | *Arcobacter lekithochrous* |  |  |
| Arcobacter_marinus_JCM15502.fasta | *Arcobacter marinus* strain JCM 15502 chromosome, complete genome |  |  |
| Arcobacter_marinusJCM15502.fasta | *Arcobacter marinus* strain JCM 15502 chromosome, complete genome |  |  |
| Arcobacter_molluscorumLMG25693CECT7696.fasta | *Arcobacter molluscorum* LMG 25693 strain CECT 7696 chromosome, complete genome |  |  |
| Arcobacter_mytili_LMG24559.fasta | *Arcobacter mytili* LMG 24559 chromosome, complete genome |  |  |
| Arcobacter_mytiliLMG24559CECT7386.fasta | *Arcobacter mytili* |  |  |
| Arcobacter_nitrofigilis_DSM7299.fasta | *Arcobacter nitrofigilis* DSM 7299, complete genome |  |  |
| Arcobacter_pacificusLMG26638.fasta | *Arcobacter pacificus* strain LMG 26638 chromosome, complete genome |  |  |
| Arcobacter_skirrowii17-1201-3.fasta | *Arcobacter skirrowii* |  |  |
| Arcobacter_skirrowii17-1201-4.fasta | *Arcobacter skirrowii* |  |  |
| Arcobacter_skirrowii17-1206-2.fasta | *Arcobacter skirrowii* |  |  |
| Arcobacter_skirrowiiCCUG10374.fasta | *Arcobacter skirrowii* CCUG 10374 chromosome, complete genome |  |  |
| Arcobacter_suisCECT7833.fasta | *Arcobacter suis* CECT 7833 chromosome, complete genome |  |  |
| Arcobacter_thereius440.fasta | *Arcobacter thereius* |  |  |
| Arcobacter_thereius452.fasta | *Arcobacter thereius* |  |  |
| Arcobacter_thereiusDU22.fasta | *Arcobacter thereius* |  |  |
| Arcobacter_thereiusLMG24486.fasta | *Arcobacter thereius* LMG 24486 chromosome, complete genome |  |  |
| Arcobacter_trophiarumLMG25534.fasta | *Arcobacter trophiarum* LMG 25534 chromosome, complete genome |  |  |
| Arcobacter_venerupis_strainCECT7836.fasta | *Arcobacter venerupis* |  |  |
